# Variations à la Fourier-Weyl-Wigner on Quantizations of the Plane and the Half-Plane

**DOI:** 10.3390/e20100787

**Published:** 2018-10-13

**Authors:** Hervé Bergeron, Jean-Pierre Gazeau

**Affiliations:** 1 ISMO, UMR 8214 CNRS, Université Paris-Sud, 91405 Orsay, France; 2 APC, UMR 7164 CNRS, Université Paris Diderot, Sorbonne Paris Cité, 75205 Paris, France

**Keywords:** Weyl-Heisenberg group, affine group, Weyl quantization, Wigner function, covariant integral quantization

## Abstract

Any quantization maps linearly function on a phase space to symmetric operators in a Hilbert space. Covariant integral quantization combines operator-valued measure with the symmetry group of the phase space. *Covariant* means that the quantization map intertwines classical (geometric operation) and quantum (unitary transformations) symmetries. *Integral* means that we use all resources of integral calculus, in order to implement the method when we apply it to singular functions, or distributions, for which the integral calculus is an essential ingredient. We first review this quantization scheme before revisiting the cases where symmetry covariance is described by the Weyl-Heisenberg group and the affine group respectively, and we emphasize the fundamental role played by Fourier transform in both cases. As an original outcome of our generalisations of the Wigner-Weyl transform, we show that many properties of the Weyl integral quantization, commonly viewed as optimal, are actually shared by a large family of integral quantizations.

## 1. Introduction: A Historical Overview

More than one century after the publication by Fourier of his “Théorie analytique de la chaleur” [1,2], the Fourier transform revealed its tremendous importance at the advent of quantum mechanics with the setting of its specific formalism, especially with the seminal contributions of Weyl (1927) [3] on phase space symmetry, and Wigner (1932) [4] on phase space distribution. The phase space they were concerned with is essentially the Euclidean plane $R^{2}=\left\{\left(q,p\right)\;,\;q,p,\in R\right\}$, *q* (mathematicians prefer to use *x*) for *position* and *p* for *momentum*. It is the phase space for the motion on the line and its most immediate symmetry is translational invariance: no point is privileged and so every point can be chosen as the origin. Non-commutativity relation $\left[Q,P\right]=i\hbar I_{H}$ between the self-adjoint quantum position *Q* and momentum *P*, the QM key stone, results from this symmetry through the Weyl projective unitary irreducible representation U [5] of the abelian group $R^{2}$ in some separable Hilbert space H,

(1) $$R^{2}∋\left(q,p\right)\mapsto U\left(q,p\right)=e^{\frac{i}{\hbar }\left(pQ-qP\right)}\;,\;U\left(q,p\right)\;U\left(q^{\prime },p^{\prime }\right)=e^{-\frac{i}{2\hbar }(qp^{\prime }-q^{\prime }p}\;U\left(q+q^{\prime },p+p^{\prime }\right)\;$$

or equivalently the true representation of the so-called Weyl-Heisenberg group, central extension with parameter ϑ of the above one,

(2) $$R\times R^{2}∋\left(\vartheta ,q,p\right)\mapsto U_{WH}\left(\vartheta ,q,p\right)=e^{i\vartheta /\hbar }U\left(q,p\right)\;.$$

In 1932, Wigner introduced his function (or quasidistribution) to study quantum corrections to classical statistical mechanics, originally in view of associating the wavefunction ψ(x), i.e., the pure state $\rho_{\psi }=\left|\psi \rangle \langle \psi \right|$, with a probability distribution in phase space. It is a Fourier transform, up to a constant factor, for all spatial autocorrelation functions of ψ(x):

(3) $$W_{\rho_{\psi }}\left(q,p\right)=2\int_{-\infty }^{+\infty }dx\;\bar{\psi (q+x)}\;\psi \left(q-x\right)\;e^{\frac{2i}{\hbar }px}=\mathrm{tr}\left(U\left(q,p\right)2PU^{†}\left(q,p\right)\rho_{\psi }\right)\;.$$

The alternative expression using in the above the parity operator (Pψ)(x)=ψ(−x) [6] allows us to extend this transform to any density operator ρ, and in fact to any traceclass operator *A* in H

(4) $$A\mapsto W_{A}\left(q,p\right)=\mathrm{tr}\left(U\left(q,p\right)2PU^{†}\left(q,p\right)A\right)\;.$$

One of the most attractive aspects of the above Wigner transform is that it is one-to-one. The inverse is precisely the Weyl quantization, more precisely the integral Weyl-Wigner quantization, defined as the map (with ℏ=1)

(5) $$f\left(q,p\right)\mapsto A_{f}=\int_{R^{2}}\frac{dq\;dp}{2\pi }\;f\left(q,p\right)\;U\left(q,p\right)2PU^{†}\left(q,p\right)=\int_{R^{2}}\frac{dq\;dp}{2\pi }U\left(q,p\right)\;\bar{F_{s}}\left[f\right]\left(q,p\right)\;.$$

Hence, $W_{A_{f}}\left(q,p\right)=f\left(q,p\right)$, with mild conditions on *f*. In the second expression of the Weyl-Wigner quantization (5) is introduced the dual of the symplectic Fourier transform. The latter is defined as

(6) $$F_{s}\left[f\right]\left(q,p\right)=\int_{R^{2}}\frac{dq\;dp}{2\pi }\;e^{-i(qp^{\prime }-q^{\prime }p)}\;f\left(q^{\prime },p^{\prime }\right)\;.$$

It is involutive, $F_{s}\left[F_{s}\left[f\right]\right]=f$ like its dual defined as $\bar{F_{s}}\left[f\right]\left(q,p\right)=F_{s}\left[f\right]\left(-q,-p\right)$.

Hence, we observe that the Fourier transform lies at the heart of the above interplay of Weyl and Wigner approaches. Please note that both the maps (46) and (5) allow one to set up a *quantum mechanics in phase space*, as was developed at a larger extent in the 1940s by Groenewold [7] and Moyal [8]. This feature became so popular that it led some people to claim that if one seeks a single consistent quantization procedure mapping functions on the classical phase space to operators, the Weyl quantization is the “best” option. Actually, we will see below that this claimed preponderance should be somewhat attenuated, for various reasons.

The organisation of the paper is as follows. In Section 2 we give a general presentation of what we call covariant integral quantization associated with a Lie group, and its *semi-classical* side. The content of this section should be viewed as a shortened reiteration of a necessary material present in previous publications by one of or both the authors, essentially [9,10,11,12,13]. The original content of the paper is found in the next sections, namely the fact that many properties of the Weyl integral quantization, commonly viewed as optimal, are actually shared by a large family of integral quantizations. In Section 3 we revisit the Weyl-Heisenberg symmetry and the related Wigner-Weyl transform and Wigner function by inserting in their integral definition a kernel which allows to preserve one of their fundamental properties, the one-to-one character of the corresponding quantization. In Section 4 we devote a similar study to the case of the half-plane, for which the affine symmetry replaces the translational symmetry, and we compare our results with some previous works. We summarize the main points of the content in Section 5. Detailed proofs of two of our results are given in Appendix A.

## 2. Covariant Integral Quantization: A Summary

Integral quantization [9,10,11,12,13] is a generic name for approaches to quantization based on operator-valued measures. It includes the so-called Berezin-Klauder-Toeplitz quantization, and more generally coherent state quantization [10,14,15]. The integral quantization framework includes as well quantizations based on Lie groups. In the sequel we will refer to this case as *covariant integral quantization*. We mentioned in the introduction its most famous example, namely the covariant integral quantization based on the Weyl-Heisenberg group (WH), like Weyl-Wigner [3,6,16,17,18] and (standard) coherent states quantizations [14]. It is well established that the WH group underlies the canonical commutation rule, a paradigm of quantum physics. However, one should be aware that there is a world of quantizations that follow this rule [9,13]. Another basic example of covariant integral quantization concerns the half-plane viewed as the phase space for the motion on the half-line. The involved Lie group is the group of affine transformations x↦(q,p)·x:=x/q+p, q>0, of the real line [9,11]. The latter has been proven essential in a series of recent works devoted to quantum cosmology [19,20,21,22,23]. Let us notice that the affine group and related coherent states were also used for the quantization of the half-plane in works by J. R. Klauder, although from a different point of view (see [24,25,26] with references therein).

### 2.1. General Settings

We first proceed with a necessary repetition of the material needed to understand the method and found in the previously quoted [9,10,11,12,13]. Let *X* be a set equipped with some structures, e.g., measure, topology, manifold, etc. In this paper *X* will be viewed as a phase space for a mechanical system. Let C(X) be a vector space of complex-valued functions f(x) on *X*, defined through some functional or distributional constraints, and viewed here as classical observables. A quantization of elements of C(X) is a linear map $Q:f\in C\left(X\right)\mapsto Q\left(f\right)\equiv A_{f}\in A\left(H\right)$ to a vector space A(H) of linear operators on some Hilbert space H. Furthermore this map must fulfill the following conditions:

(i) To f=1 there corresponds $A_{f}=I_{H}$, where $I_{H}$ is the identity in H,
(ii) To a real function f∈C(X) there corresponds a(n) (essentially) self-adjoint operator $A_{f}$ in H.

From a physical point of view it will be necessary to add to this minimal material an interpretative measurement context.

Let us now assume that X=G is a Lie group with left Haar measure dμ(g). Let $g\mapsto U_{g}$ be a unitary irreducible representation (UIR) of *G* as operators in H Let *M* be a bounded self-adjoint operator on H and let us define $U_{g}$-translations of *M* as

(7) $$M\left(g\right)=U_{g}MU_{g}^{†}\;.$$

The application of Schur’s Lemma under mild conditions allows to infer that there exists a real constant $c_{M}\in R$ such that the following resolution of the identity holds (in the weak sense of bilinear forms)

(8) $$\int_{G}M\left(g\right)\frac{d\mu (g)}{c_{M}}=I_{H}\;.$$

For instance, in the case of a square-integrable unitary irreducible representation $U:g\mapsto U_{g}$ (see Chapters 7 and 8 in [10] for details and references), let us pick a unit vector |ψ〉 for which $c_{M}=\int_{G}d\mu \left(g\right){\left|\langle \psi |U_{g}\psi \rangle \right|}^{2}<\infty$, i.e., |ψ〉 is an admissible unit vector for *U*. With M=|ψ〉〈ψ| the resolution of the identity (8) provided by the family of states $|\psi_{g}\rangle =U_{g}|\psi \rangle$ reads

(9) $$\int_{G}|\psi_{g}\rangle \langle \psi_{g}|\frac{d\mu (g)}{c_{M}}=I_{H}\;.$$

Vectors $|\psi_{g}\rangle$ are named (generalized) coherent states (or wavelets) for the group *G*.

With the resolution (8) in hand one can proceed with the integral quantization of complex-valued functions or distributions on the group *G* as follows

(10) $$f\mapsto A_{f}=\int_{G}M\left(g\right)f\left(g\right)\frac{d\mu (g)}{c_{M}}\;.$$

Of course, some conditions have to be imposed to *f* in order to ensure the existence of the operator, or *quantum observable*, $A_{f}$. With such conditions, the quantization (10) is covariant in the sense that $U_{g}A_{f}U_{g}^{†}=A_{F}$ where $F\left(g^{\prime }\right)=\left(U_{g}f\right)\left(g^{\prime }\right)=f\left(g^{-1}g^{\prime }\right)$.

To be more precise about the existence of the operator-valued integral in (10), the latter should be understood in a weak sense. Precisely, the sesquilinear form

(11) $$H∋\psi_{1},\psi_{2}\mapsto B_{f}\left(\psi_{1},\psi_{2}\right)=\int_{G}\langle \psi_{1}|M_{g}|\psi_{2}\rangle f\left(g\right)\frac{d\mu (g)}{c_{M}}\;,$$

is assumed to be defined on a dense subspace of H. If *f* is a complex bounded function, $B_{f}$ is a bounded sesquilinear form, and from the Riesz lemma we deduce that there exists a unique bounded operator $A_{f}$ associated with $B_{f}$. If *f* is real and semi-bounded, and if *M* is a positive operator, Friedrich’s extension of $B_{f}$ ([27], Thm. X23) univocally defines a self-adjoint operator. However, if *f* is real but not semi-bounded, there is no natural choice for a self-adjoint operator associated with $B_{f}$. In this case, one can consider directly the symmetric operator $A_{f}$ enabling us to obtain a possible self-adjoint extension (an example of this kind of mathematical study is presented in [28]).

### 2.2. Semi-Classical Framework With Probabilistic Interpretation

Integral quantization allows to develop what is commonly viewed as a semi-classical analysis/interpretation of quantum observables. If M=ρ and $\tilde{\rho }$ are two non-negative (“density operator”) unit trace operators, we obtain the classical-like expectation value formula

(12) $$\mathrm{tr}\left(\tilde{\rho }A_{f}\right)=\int_{G}f\left(g\right)w\left(g\right)\frac{d\mu (g)}{c_{M}}\;.$$

Indeed, resolution of the identity, non-negativeness and unit-trace conditions imply that $w\left(g\right)=\mathrm{tr}(\tilde{\rho }\;\rho \left(g\right))\geq 0$ is, up to the coefficient $c_{M}$, a classical probability distribution on the group. Moreover, we consider the map

(13) $$f\mapsto \overset{ˇ}{f}\left(g\right)=\int_{G}\mathrm{tr}\left(\tilde{\rho }\left(g\right)\;\rho \left(g^{\prime }\right)\right)\;f\left(g^{\prime }\right)\frac{d\mu (g)}{c_{M}}.$$

as a generalization of Berezin or heat kernel or Segal-Bargmann transforms [29] on *G*. Given *f*, the new function $\overset{ˇ}{f}$ is called lower or covariant symbol of the operator $A_{f}$. It may be viewed as one of its semi-classical representations.

In the case of coherent states $|\psi_{g}\rangle$ (i.e., M=ρ=|ψ〉〈ψ|), Equation (12) reads

(14) $$\mathrm{tr}\left(\tilde{\rho }A_{f}\right)=\int_{G}f\left(g\right)\;\langle \psi_{g}|\tilde{\rho }|\psi_{g}\rangle \frac{d\mu (g)}{c_{M}}\;,$$

where $w\left(g\right)=\langle \psi_{g}|\tilde{\rho }|\psi_{g}\rangle \geq 0$ is viewed here as a classical probability distribution on the group (up to the coefficient $c_{M}$). Similarly assuming $\tilde{\rho }=|\tilde{\psi }\rangle \langle \tilde{\psi }|$, the lower symbol $\overset{ˇ}{f}\left(g\right)$ involved in (13) reads

(15) $$\overset{ˇ}{f}\left(g\right)=\int_{G}{\left|\langle {\tilde{\psi }}_{g}|\psi_{g^{\prime }}\rangle \right|}^{2}f\left(g^{\prime }\right)\frac{d\mu (g^{\prime })}{c_{M}}$$

### 2.3. Semi-Classical Picture Without Probabilistic Interpretation

A semi-classical framework similar to (13) can be also developed if the operators *M* and $\tilde{M}$ are not positive:

(16) $$f\mapsto \overset{ˇ}{f}\left(g\right)=\mathrm{tr}\left(\tilde{M}\left(g\right)A_{f}\right)=\int_{G}\mathrm{tr}\left(\tilde{M}\left(g\right)\;M\left(g^{\prime }\right)\right)\;f\left(g^{\prime }\right)\frac{d\mu (g^{\prime })}{c_{M}}$$

Then the probabilistic interpretation is lost in general due to the loss of positiveness of the map $g^{\prime }\mapsto \mathrm{tr}\left(\tilde{M}\left(g\right)M\left(g^{\prime }\right)\right)$. However, in some special cases Equation (16) allows one to obtain an inverse of the quantization map (10). Namely for special pairs $\left(M,\tilde{M}\right)$ we obtain

(17) $$\mathrm{tr}\left(\tilde{M}\left(g\right)\;A_{f}\right)=f\left(g\right)$$

In the sequel we analyze different examples of this kind in the case of the quantization of the plane (Weyl-Heisenberg group) and the half-plane (affine group).

## 3. Quantization of the Plane: Generalizations of the Wigner-Weyl Transform

### 3.1. The Group Background

Let us first recall some definitions with more details about the Weyl-Heisenberg (WH) group $G_{WH}$, that we have already mentioned in the introduction. More details can be found for instance in [10,13]. It is a central extension of the group of translations of the two-dimensional euclidean plane. In classical mechanics the latter is viewed as the phase space for the motion of a particle on the real line. The UIR we are concerned with is the unitary representation of $G_{WH}$, acting in some separable Hilbert space H, which integrates the canonical commutation rule (CCR) of quantum mechanics, $\left[Q,P\right]=i\hbar I_{H}$. Forgetting about physical dimensions (ℏ=1), an arbitrary element *g* of $G_{WH}$ is of the form

(18) $$g=\left(\vartheta ,q,p\right),\;\vartheta \in R,\;\left(q,p\right)\in R^{2},$$

with multiplication law

(19) $$g_{1}g_{2}=\left(\vartheta_{1}+\vartheta_{2}+\zeta \left[\left(q_{1},p_{1}\right),\left(q_{2},p_{2}\right)\right],q_{1}+q_{2},p_{1}+p_{2}\right)\;,$$

where ζ is the multiplier function $\zeta \left[\left(q_{1},p_{1}\right),\left(q_{2},p_{2}\right)\right]=\frac{1}{2}\left(p_{1}q_{2}-p_{2}q_{1}\right)$. Any infinite dimensional UIR $U_{WH}^{\lambda }$ of $G_{WH}$ is characterized by a real number λ≠0 (in addition, there are also degenerate, one-dimensional, UIR’s corresponding to λ=0, but they are irrelevant here). These UIR’s may be realized on the same Hilbert space H, as the one carrying an irreducible representation of the CCR:

(20) $$U_{WH}^{\lambda }\left(\vartheta ,q,p\right)=e^{i\lambda \vartheta }U^{\lambda }\left(q,p\right)=e^{i\lambda (\theta -qp/2)}e^{i\lambda pQ}e^{-i\lambda qP}\;.$$

If $H=L^{2}\left(R,dx\right)$ corresponding to the spectral decomposition $Q=\int_{R}x\;|x\rangle \langle x|\;dx$ of the essentially self-adjoint position operator *Q*, the action of $U_{WH}^{\lambda }$ reads as

(21) $$\left(U_{WH}^{\lambda }\left(\vartheta ,q,p\right)\phi \right)\left(x\right)=e^{i\lambda \vartheta }e^{i\lambda p(x-q/2)}\phi \left(x-q\right),\;\phi \in L^{2}\left(R,dx\right)\;.$$

Thus, the three operators $I_{H}$, *Q*, *P* appear as the generators of this representation and are realized as:

(22) $$\left(Q\phi \right)\left(x\right)=x\phi \left(x\right),\;\left(P\phi \right)\left(x\right)=-\frac{i}{\lambda }\phi^{\prime }\left(x\right),\;\left[Q,P\right]=\frac{i}{\lambda }I_{H}\;.$$

For our purpose we take λ=1/ℏ=1 and simply write $U_{WH}$ for the corresponding representation.

### 3.2. Hyperbolic W-H Covariant Integral Quantization

#### 3.2.1. General Settings

We investigate special cases of the Weyl-Heisenberg covariant integral quantization that have remarkable properties. They are included in our general framework as a special case. Namely let us choose some function $F\in L^{1}\left(R,dx\right)$ and define its Fourier transform $\hat{F}$ as

(23) $$\hat{F}\left(\omega \right)=\int_{R}F\left(u\right)e^{-i\omega u}\;du\;.$$

This framework will be extended to distributions when necessary. We define the operator $P_{0}^{\left(F\right)}$ (corresponding to the operator (denoted by *M* in Section 2.1) as the Weyl transform of $\hat{F}$:

(24) $$P_{0}^{\left(F\right)}=\int_{R^{2}}\frac{dqdp}{2\pi }\hat{F}\left(qp\right)\;e^{i(pQ-qP)}\;.$$

The associate quantization is named *hyperbolic* because of this special dependence through a function of qp. The operator $P_{0}^{\left(F\right)}$ is bounded if $F\in L^{1}\left(R,{\left|u^{2}-1/4\right|}^{-1/2}du\right)$ (see Appendix A for the proof). The main interest of this choice at the physical level is that all quantizations of this kind involve solely the Planck constant *ℏ* as a dimensional parameter. In fact, *ℏ* can be restored as follows

(25) $$P_{0}^{\left(F\right)}=\int_{R^{2}}\frac{dqdp}{2\pi \hbar }\hat{F}\left(qp/\hbar \right)\;e^{i(pQ-qP)/\hbar }\;.$$

The already mentioned canonical Wigner-Weyl transform or the Born-Jordan quantization [30,31,32] are special cases, but the above generalisation of the latter offers a large freedom in the choice of *F* with no need for introducing extra dimensional parameters.

In terms of the Dirac *kets* |x〉 such that Q|x〉=x|x〉, the kernel $\langle x|P_{0}^{\left(F\right)}|y\rangle$ reads as:

(26) $$\langle x|P_{0}^{\left(F\right)}|y\rangle =\frac{1}{\left|x-y\right|}\int_{R}\frac{du}{2\pi }\hat{F}\left(u\right)\exp\left(iu\frac{x+y}{2(x-y)}\right)$$

which gives

(27) $$\langle x|P_{0}^{\left(F\right)}|y\rangle =\frac{1}{\left|x-y\right|}F\left(\frac{x+y}{2(x-y)}\right)\;.$$

The bounded operator $P_{0}^{\left(F\right)}$ is self-adjoint if *F* verifies the hilbertian symmetry $\bar{F(u)}=F\left(-u\right)$. We assume this condition to be fulfilled in the sequel.

The kernel of the operator $P_{q,p}^{\left(F\right)}$ corresponding to the WH transported operators M(g) as in Equation (7) reads

(28) $$\langle x|P_{q,p}^{\left(F\right)}|y\rangle =\frac{1}{\left|x-y\right|}F\left(\frac{x+y-2q}{2(x-y)}\right)e^{ip(x-y)}\;.$$

While the variable *p* appears in this formula as the Fourier reciprocal variable, the variable *q* appears as a translation parameter from the *arithmetic* mean of the variables *x* and *y*. Such an observation will take its real importance when we will deal with the affine symmetry in the next part of this paper.

#### 3.2.2. Resolution of the Identity

From the Weyl-Heisenberg covariance and Schur’s lemma, we obtain the resolution of unity as

(29) $$\int_{R^{2}}\frac{dq\;dp}{2\pi }P_{q,p}^{\left(F\right)}=c\;I_{H}$$

where $c=\int_{R}F\left(u\right)du$. Therefore we assume in the sequel $\int_{R}F\left(u\right)du=1$.

At this point it is valuable to give a direct proof of (29). Due to the polarization identity, it is sufficient to prove that for any ψ∈H:

(30) $$\int_{R^{2}}\frac{dq\;dp}{2\pi }\langle \psi |P_{q,p}^{\left(F\right)}|\psi \rangle =c\langle \psi |\psi \rangle \;.$$

First

(31) $$\langle \psi |P_{q,p}^{\left(F\right)}|\psi \rangle =\int_{R^{2}}dx\;dy\;\bar{\psi (x)}\;\psi \left(y\right)\;\frac{1}{\left|x-y\right|}\;F\left(\frac{x+y-2q}{2(x-y)}\right)e^{ip(x-y)}\;.$$

By performing the change of variables X=(x+y)/2, z=x−y, we obtain

(32) $$\langle \psi |P_{q,p}^{\left(F\right)}|\psi \rangle =\int_{R^{2}}dX\;dz\;\bar{\psi (X+z/2)}\;\psi \left(X-z/2\right)\frac{1}{\left|z\right|}F\left(\frac{X-q}{z}\right)e^{ipz}\;.$$

Then we keep *z* and we change *X* in u=(X−q)/z. This leads to

(33) $$\langle \psi |P_{q,p}^{\left(F\right)}|\psi \rangle =\int_{R^{2}}du\;dz\;F\left(u\right)\;e^{ipz}\;\bar{\psi (q+(u+1/2)z)}\;\psi \left(q+\left(u-1/2\right)z\right)\;.$$

We remark that this equation is in fact a generalization of the Wigner function. The latter is recovered with F(u)=δ(u). In this sense, the function *F* is a Cohen kernel [33,34], but its interpretation in the present quantization context is different of the role it was given by this author and others, like [35]. Now the integral over *p* gives

(34) $$\int_{R}\frac{dp}{2\pi }\langle \psi |P_{q,p}^{\left(F\right)}|\psi \rangle =\int_{R}du\;F\left(u\right){\left|\psi \left(q\right)\right|}^{2}\;.$$

and finally

(35) $$\int_{R^{2}}\frac{dq\;dp}{2\pi }\langle \psi |P_{q,p}^{\left(F\right)}|\psi \rangle =\langle \psi |\psi \rangle \int_{R}du\;F\left(u\right)\;.$$

Assuming ∫duF(u)=1, we recover the resolution of the identity.

#### 3.2.3. Covariant Quantization and Properties

The *F*-dependent quantization map $f\mapsto A_{f}^{\left(F\right)}$ is defined as

(36) $$f\mapsto A_{f}^{\left(F\right)}=\int_{R^{2}}\frac{dqdp}{2\pi }f\left(q,p\right)\;P_{q,p}^{\left(F\right)}$$

The usual Wigner-Weyl kernel corresponds to the distribution choice F(x)=δ(x) and it is, therefore, singular with respect to the functional framework. The case of Born-Jordan corresponds to the choice of the indicator function $F\left(u\right)=1_{\left[-1/2,1/2\right]}\left(u\right)$. The map $f\mapsto A_{f}^{\left(F\right)}$ is such that whatever *F* (under the above conditions)

(37) $$A_{q}^{\left(F\right)}=Q\;\mathrm{and}\;A_{p}^{\left(F\right)}=P\;,$$

and more generally,

(38) $$A_{f(q)}^{\left(F\right)}=f\left(Q\right)\;\mathrm{and}\;A_{f(p)}^{\left(F\right)}=f\left(P\right)\;.$$

Therefore, by linearity any classical Hamiltonian $h\left(q,p\right)=\frac{1}{2m}p^{2}+V\left(q\right)$ is mapped into the quantum Hamiltonian $H=\frac{1}{2m}P^{2}+V\left(Q\right)$ that has the same form. Moreover, with the same conditions on *F*, we have

(39) $$A_{qp}^{\left(F\right)}=\frac{1}{2}\left(QP+PQ\right)+c,\;\mathrm{with}\;c=-i\int_{R}uF\left(u\right)du\;.$$

The constant *c* is real due to the condition $\bar{F(u)}=F\left(-u\right)$. If F(u) is real then c=0.

**Remark** **1.** *Different quantizations generated by different F cannot be distinguished only using the most common operators involved in non-relativistic quantum mechanics (and corresponding to observables that can be really measured). Therefore there is no reason to privilege a specific one (for example the canonical one).*

#### 3.2.4. Trace Formula

Let us rewrite (28) as:

(40) $$P_{q,p}^{\left(F\right)}=\int_{R^{2}}dxdy\;\frac{1}{\left|x-y\right|}F\left(\frac{x+y-2q}{2(x-y)}\right)e^{ip(x-y)}|x\rangle \langle y|\;.$$

Using the same kind of transformations as the ones used for the resolution of the identity we have (formally):

(41) $$P_{q,p}^{\left(F\right)}=\int_{R^{2}}du\;dz\;F\left(u\right)\;e^{ipz}|q+\left(u+1/2\right)z\rangle \;\langle q+\left(u-1/2\right)z|\;.$$

Then (still formally)

(42) $$\mathrm{tr}\;P_{q,p}^{\left(F\right)}=\int_{R^{2}}du\;dz\;F\left(u\right)\;e^{ipz}\;\delta \left(z\right)=1\;.$$

For two different functions *F* and *G* we obtain the trace formula:

(43) $$\mathrm{tr}\left(P_{q,p}^{\left(F\right)}P_{q^{\prime },p^{\prime }}^{\left(G\right)}\right)=\int_{R}\frac{dz}{\left|z\right|}\;e^{-i(p-p^{\prime })z}\left(F*G\right)\left(\frac{q-q^{\prime }}{z}\right)\;.$$

where F∗G is the convolution product of *F* and *G*.

### 3.3. Invertible W-H Covariant Integral Quantization: Generalization of the Wigner-Weyl Transform

#### 3.3.1. General Settings

Let us examine the case for which (43) gives the equation F∗G=δ. Please note that such an equation has no solution with a pair of summable functions. In this case, we have

(44) $$\mathrm{tr}\;\left(P_{q,p}^{\left(F\right)}P_{q^{\prime },p^{\prime }}^{\left(G\right)}\right)=2\pi \;\delta \left(q-q^{\prime }\right)\;\delta \left(p-p^{\prime }\right)\;.$$

Therefore if *F* possesses a convolution inverse *G*, the quantization map is invertible. Indeed if *G* is the inverse of convolution of *F* then

(45) $$\mathrm{tr}\left(P_{q,p}^{\left(G\right)}A_{f}^{\left(F\right)}\right)=f\left(q,p\right)\;.$$

In this regard, the Wigner-Weyl case is *trivial* in the sense that F=δ is its own inverse and therefore the Wigner-Weyl quantization map is inverted with the same operator. Furthermore since δ is a distribution, the Wigner-Weyl choice is in fact *singular* within this functional framework. Therefore using a true function *F* can be viewed as a *regularization*. However, this regularization in the quantization map has a cost: the inverse map (if it exists) is more singular than a pure δ.

In the case of Born-Jordan the Fourier transform of the indicator function F(u) is $\hat{F}\left(k\right)=\frac{\sin(k/2)}{k/2}$ that possesses simple zeros on the real axis. Whence the convolution inverse of *F* only exists in a distribution sense as a series of principal values.

#### 3.3.2. Generalized Wigner Functions

Given a function *F*, we now define the *generalized Wigner function* of an operator *A* as

(46) $$W_{A}^{\left(F\right)}\left(q,p\right)=\mathrm{tr}\left(P_{q,p}^{\left(F\right)}A\right)\;.$$

If *A* is the pure state |ψ〉〈ψ|, this function reads

(47) $$\begin{array}{cc} W_{\psi }^{\left(F\right)}\left(q,p\right) & \equiv W_{\left|\psi \rangle \langle \psi \right|}^{\left(F\right)}\left(q,p\right)=\langle \psi |P_{q,p}^{\left(F\right)}|\psi \rangle \end{array}$$

(48) $$\begin{array}{c} =\int_{R^{2}}du\;dz\;F\left(u\right)\;e^{ipz}\;\bar{\psi (q+(u+1/2)z)}\;\psi \left(q+\left(u-1/2\right)z\right)\;. \end{array}$$

The standard Wigner function corresponds to $W_{\psi }^{\left(\delta \right)}\left(q,p\right)$. All functions $W_{\psi }^{\left(F\right)}\left(q,p\right)$ share the same marginal properties. Namely the functions $q\mapsto {\left(2\pi \right)}^{-1}\int dpW_{\psi }^{\left(F\right)}\left(q,p\right)$ and $p\mapsto {\left(2\pi \right)}^{-1}\int dqW_{\psi }^{\left(F\right)}\left(q,p\right)$ are the exact quantum probability distributions for position and momentum. This is a direct consequence of (38). Furthermore, because of the invertible character of the corresponding Wigner-Weyl transform, i.e.,

(49) $$W_{A_{f}}^{\left(\delta \right)}\left(q,p\right):=\mathrm{tr}\left(P_{q,p}^{\left(\delta \right)}A_{f}\right)=f\left(q,p\right)\;,$$

we have

(50) $$\left|\psi \rangle \langle \psi \right|=\int_{R^{2}}\frac{dq^{\prime }\;dp^{\prime }}{2\pi }W_{\psi }^{\left(\delta \right)}\left(q^{\prime },p^{\prime }\right)P_{q^{\prime },p^{\prime }}^{\left(\delta \right)}\;.$$

Therefore

(51) $$W_{\psi }^{\left(F\right)}\left(q,p\right)=\int_{R^{2}}\frac{dq^{\prime }\;dp^{\prime }}{2\pi }W_{\psi }^{\left(\delta \right)}\left(q^{\prime },p^{\prime }\right)\;\mathrm{tr}\left(P_{q,p}^{\left(F\right)}P_{q^{\prime },p^{\prime }}^{\left(\delta \right)}\right)\;.$$

Using (43) we obtain

(52) $$W_{\psi }^{\left(F\right)}=W_{\psi }^{\left(\delta \right)}*\Lambda \left(F\right)\;.$$

where ∗ holds for the 2d-convolution product with the measure $\frac{dq\;dp}{2\pi }$ and

(53) $$\Lambda \left(F\right)\left(q,p\right)=\tilde{F}\left(qp\right),\;\mathrm{with}\;\tilde{F}\left(\omega \right)=\int_{R}\frac{d\alpha }{\left|\alpha \right|}e^{-i\;\omega /\alpha }\;F\left(\alpha \right)$$

**Remark** **2.**

- *The function Λ(F) only depends on the variable qp. Therefore it cannot belong to some $L^{r}$ space on the plane. Hence, the convolution product involved in (52) should be understood in general in the distribution sense.*
- *The function $\tilde{F}$ is defined as an integral only if F belongs to $L^{1}{\left(R,|\alpha \right|}^{-1}d\alpha )$. In other cases an extension in the distribution framework is needed.*
- *An interesting question concerns the positiveness of $W_{\psi }^{\left(F\right)}$. In the genuine Wigner-Weyl case (F=δ), Hudson theorem [36] asserts that only gaussian states ψ lead to positive Wigner functions $W_{\psi }^{\left(\delta \right)}\left(q,p\right)$, and so the latter can be interpreted as probability densities on phase space. Beyond the pure Gaussian case, see for instance [37]. The problem now is to formulate a generalized version of the Hudson theorem (involving maybe a different family of states) for the generalized Wigner function $W_{\psi }^{\left(F\right)}$). In other words, for a given state ψ, is it possible to “build” a function F such that the corresponding Wigner function $W_{\psi }^{\left(F\right)}$ is positive?*

#### 3.3.3. Examples of Invertible Map

In the following lines, we give an explicit example of invertible map, dependent on two strictly positive parameters α and β and that includes the Wigner-Weyl solution as a special case (this example was found through the use of Fourier transform). Let us define $F_{\alpha ,\beta }$ as

(54) $$F_{\alpha ,\beta }\left(x\right)=\alpha^{4}\delta \left(x\right)+\frac{1}{2}\alpha \beta \left(1-\alpha^{4}\right)e^{-\alpha \beta |x|}\;.$$

Obviously we have $\bar{F(x)}=F\left(-x\right)$ (in the distribution sense), and formally ∫F(x)dx=1. Taking into account the elementary result for a,b>0:

(55) $$e^{-a|x|}*e^{-b|x|}=\frac{2}{b^{2}-a^{2}}\left(be^{-a|x|}-ae^{-b|x|}\right)\;,$$

we find that a convolution inverse of $F_{\alpha ,\beta }$ is $F_{\alpha^{\prime },\beta^{\prime }}$ with $\alpha^{\prime }=1/\alpha$ et $\beta^{\prime }=\beta \alpha^{-2}$. The Wigner-Weyl case corresponds to the degenerate case $F_{1,\beta }\left(x\right)=\delta \left(x\right)$.

## 4. Quantization of the Half-Plane With the Affine Group: Wigner-Weyl-Like Scheme

### 4.1. The Group Background

The half-plane is defined as $\Pi_{+}=\left\{\left(q,p\right)\;|\;q>0,p\in R\right\}$. Equipped with the law

(56) $$\left(q,p\right)\left(q^{\prime },p^{\prime }\right)=\left(qq^{\prime },p+\frac{p^{\prime }}{q}\right)\;,$$

$\Pi_{+}$ is viewed as the affine group Aff${}_{+}\left(R\right)$ of the real line. The left invariant measure is dμ(q,p)=dqdp. Besides a trivial one, the affine group possesses two nonequivalent square integrable UIR’s. Equivalent realizations of one of them, say, *U*, are carried by Hilbert spaces $L^{2}\left(R_{+},dx/x^{\mu }\right)$. Nonetheless these multiple possibilities do not introduce noticeable differences. Therefore we choose in the sequel μ=0, and denote $H=L^{2}\left(R_{+},dx\right)$. The UIR of Aff${}_{+}\left(R\right)$, when expressed in terms of the (dimensionless) phase-space variables (q,p), acts on H as

(57) $$U_{q,p}\psi \left(x\right)=\frac{1}{\sqrt{q}}e^{ipx}\psi \left(x/q\right)\;.$$

We define the (essentially) self-adjoint operator *Q* on H as the multiplication operator (Qϕ)(x)=xϕ(x) and the symmetric operator *P* as $\left(P\phi \right)\left(x\right)=-i\phi^{\prime }\left(x\right)$. Let us note that *P* has no self-adjoint extension in H [27].

### 4.2. Wigner-Weyl-Like Covariant Affine Quantization

#### General Settings

In the continuation of the procedure exposed in the previous sections, we now investigate special cases of affine covariant integral quantization that leads to remarkable properties. They are analogous to the Wigner-Weyl transform on the plane. As for the plane, the interest of these cases on the physical level is that if we restore physical dimensions for *q* or *x* (length) and *p* (momentum) they only include the Planck constant as a dimensional parameter. The freedom of the quantization map lies again in the choice of a pure mathematical function *F*. This section generalizes Wigner-like and Weyl-like aspects of affine covariant quantization presented in [11] by introducing families of invertible mappings that look like the Wigner-Weyl case in the plane (see the discussion below).

In this affine context, we define the operators $P_{q,p}^{\left(F\right)}$, $\left(q,p\right)\in \Pi_{+}$, dependent on a possibly complex function $F:R^{+}∋u\mapsto F\left(u\right)\in C$, by their kernel $\langle x|P_{q,p}^{\left(F\right)}|y\rangle$ in the generalized basis |x〉, x≥0, such that Q|x〉=x|x〉:

(58) $$\langle x|P_{q,p}^{\left(F\right)}|y\rangle =\delta \left(\sqrt{xy}-q\right)F\left(\sqrt{x/y}\right)e^{ip(x-y)}\;,$$

Note the alternative expression, $\delta \left(\sqrt{xy}-q\right)=\left(2q/x\right)\delta \left(y-q^{2}/x\right)$.

It is easy to verify that the covariance with respect to the affine group holds true. If needed, we remind that the presence of the Planck constant is restored by replacing $e^{ip(x-y)}$ with $\exp\left(\frac{i}{\hbar }p\left(x-y\right)\right)$.

We prove in Appendix B that the operator $P_{q,p}^{\left(F\right)}$ is bounded if the function $u\mapsto u^{2}F\left(u\right)$ is bounded. In addition, to impose the self-adjointness of $P_{q,p}^{\left(F\right)}$ we assume that *F* fulfills the symmetry: $\bar{F(x)}=F\left(1/x\right)$.

**Remark** **3.** *We already noticed that the Wigner-Weyl transform on the plane induced by the operators $P_{q,p}^{\left(\delta \right)}$ introduced in the previous section involves the arithmetic mean (x+y)/2 through $\delta (2^{-1}\left(x+y\right)-q)$. In the present case of the half-plane, its affine symmetry leads us to replace the arithmetic mean by the geometric mean $\sqrt{xy}$ appearing in $\delta (\sqrt{xy}-q)$.*

### 4.3. Resolution of the Identity

The operators $P_{q,p}^{\left(F\right)}$ defined by their kernels (58) solve the identity. Indeed, we check (formally) that

(59) $$\int_{R}\frac{dp}{2\pi }\langle x|P_{q,p}^{\left(F\right)}|y\rangle =\delta \left(x-y\right)\delta \left(x-q\right)F\left(1\right)\;,$$

and therefore

(60) $$\int_{R^{+}\times R}\frac{dqdp}{2\pi }\langle x|P_{q,p}^{\left(F\right)}|y\rangle =F\left(1\right)\delta \left(x-y\right)$$

Therefore if we impose F(1)=1 we obtain the resolution of the identity.

In the sequel we assume the function *F* fulfill both the conditions F(1)=1 and $\bar{F(x)}=F\left(1/x\right)$.

### 4.4. Affine Covariant Quantization and Properties

The *F*-dependent quantization map $f\mapsto A_{f}^{\left(F\right)}$ is defined as

(61) $$f\mapsto A_{f}^{\left(F\right)}=\int_{\Pi_{+}}\frac{dq\;dp}{2\pi }f\left(q,p\right)\;P_{q,p}^{\left(F\right)}\;.$$

This map is such that whatever *F* (under the above conditions) we have:

(62) $$A_{q}^{\left(F\right)}=Q,\;A_{p}^{\left(F\right)}=P+\frac{i}{2Q}F^{\prime }\left(1\right)\;.$$

$A_{p}$ is symmetric because $\bar{F^{\prime }\left(1\right)}=-F^{\prime }\left(1\right)$. If we impose *F* to be real, then we have F(u)=F(1/u) and then $F^{\prime }\left(1\right)=0$, therefore $A_{p}^{\left(F\right)}=P$.

More generally, whatever *F* we have the following relation which is similar to the Wigner-Weyl quantization map:

(63) $$A_{f(q)}^{\left(F\right)}=f\left(Q\right)\;.$$

Whatever *F* we have for the kinetic term $p^{2}$,

(64) $$A_{p^{2}}^{\left(F\right)}=P^{2}+\frac{iF^{\prime }\left(1\right)}{2}\left(\frac{1}{Q}P+P\frac{1}{Q}\right)-\frac{F^{\prime \prime }\left(1\right)+F^{\prime }\left(1\right)}{4Q^{2}}\;.$$

From $\bar{F^{\prime }\left(1\right)}=-F^{\prime }\left(1\right)$, and $\bar{F^{\prime \prime }\left(1\right)}=2F^{\prime }\left(1\right)+F^{\prime \prime }\left(1\right)$ one deduces that $A_{p^{2}}$ is symmetric.

If F(u) is real, then F(u)=F(1/u), and $F^{\prime }\left(1\right)=0$ (but the sign of $F^{\prime \prime }\left(1\right)$ is unspecified). It follows that

(65) $$A_{p^{2}}^{\left(F\right)}=P^{2}-\frac{F^{\prime \prime }\left(1\right)}{4Q^{2}}\;.$$

If $F^{\prime \prime }\left(1\right)<-3$ then $A_{p^{2}}^{\left(F\right)}$ has a unique self-adjoint extension on H [27,38].

We notice that at the opposite of the Wigner-Weyl case we have not in general $A_{f(p)}=f\left(P\right)$. The arbitrary choice of function *F* allows some regularization at the operator level. For example, in the case of $A_{p^{2}}^{\left(F\right)}$, an adequate choice of *F* leads to a natural unique self-adjoint extension that uniquely specifies the quantization of $p^{2}$.

#### Trace Formula

The trace of $P_{q,p}^{\left(F\right)}$ reads (formally)

(66) $$\mathrm{tr}\;P_{q,p}^{\left(F\right)}=\int_{R^{+}}dx\langle x|P_{q,p}^{\left(F\right)}|x\rangle =\int_{R^{+}}dx\delta \left(x-q\right)F\left(1\right)=F\left(1\right)=1\;.$$

Concerning the trace of the product of two different operators $P_{q,p}^{\left(F\right)}$ and $P_{q^{\prime },p^{\prime }}^{\left(G\right)}$ we successively have

(67) $$\begin{array}{cc} \mathrm{tr}\;\left(P_{q,p}^{\left(F\right)}P_{q^{\prime },p^{\prime }}^{\left(G\right)}\right) & =\int_{R^{+}\times R^{+}}dx\;dy\;\langle x|P_{q,p}^{\left(F\right)}\left|y\rangle \langle y\right|P_{q^{\prime },p^{\prime }}\left(G\right)|x\rangle \\ & =2\sqrt{qq^{\prime }}\delta \left(q-q^{\prime }\right)\int_{R^{+}}\frac{dx}{x}\exp\left(i\left(p-p^{\prime }\right)\left(x-\frac{qq^{\prime }}{x}\right)\right)F\left(\frac{x}{\sqrt{qq^{\prime }}}\right)G\left(\frac{\sqrt{qq^{\prime }}}{x}\right)\\ & =2\sqrt{qq^{\prime }}\delta \left(q-q^{\prime }\right)\int_{R^{+}}\frac{du}{u}\exp\left(i\left(p-p^{\prime }\right)\sqrt{qq^{\prime }}\left(u-1/u\right)\right)F\left(u\right)G\left(1/u\right)\;. \end{array}$$

Applying our symmetry assumption $\bar{G(x)}=G\left(1/x\right)$ we get

(68) $$\mathrm{tr}\;\left(P_{q,p}^{\left(F\right)}P_{q^{\prime },p^{\prime }}^{\left(G\right)}\right)=2\sqrt{qq^{\prime }}\delta \left(q-q^{\prime }\right)\int_{R^{+}}\frac{du}{u}\exp\left(i\left(p-p^{\prime }\right)\sqrt{qq^{\prime }}\left(u-1/u\right)\right)F\left(u\right)\bar{G(u)}$$

We now define the function $\phi :R^{+}∋u\mapsto \xi =u-1/u\in R$. We have $\phi^{\prime }\left(u\right)=1+u^{-2}$ and $u=\phi^{-1}\left(\xi \right)=\left(\xi /2\right)+\sqrt{{\left(\xi /2\right)}^{2}+1}$. Therefore

(69) $$\begin{array}{cc} \mathrm{tr}\left(P_{q,p}^{\left(F\right)}P_{q^{\prime },p^{\prime }}^{\left(G\right)}\right) & =\sqrt{qq^{\prime }}\delta \left(q-q^{\prime }\right)\int_{R}\frac{d\xi }{\xi /2+\sqrt{{\left(\xi /2\right)}^{2}+1}}\left(1+\frac{\xi }{\sqrt{\xi^{2}+4}}\right)\times \\ & \times e^{i\left(p-p^{\prime }\right)\sqrt{qq^{\prime }}\xi }F\left[\phi^{-1}\left(\xi \right)\right]\bar{G[\phi^{-1}\left(\xi \right)]}\\ & =2\sqrt{qq^{\prime }}\delta \left(q-q^{\prime }\right)\int_{R}\frac{d\eta }{\sqrt{\eta^{2}+1}}e^{2i\left(p-p^{\prime }\right)\sqrt{qq^{\prime }}\eta }F\left[\phi^{-1}\left(2\eta \right)\right]\bar{G[\phi^{-1}\left(2\eta \right)]}\;. \end{array}$$

Defining $\tilde{F}\left(\eta \right)$ (and $\tilde{G}\left(\eta \right)$) as

(70) $$\tilde{F}\left(\eta \right)=\frac{1}{{\left(\eta^{2}+1\right)}^{1/4}}F\left[\phi^{-1}\left(2\eta \right)\right]\;,$$

we finally get

(71) $$\mathrm{tr}\;\left(P_{q,p}^{\left(F\right)}P_{q^{\prime },p^{\prime }}^{\left(G\right)}\right)=2\sqrt{qq^{\prime }}\delta \left(q-q^{\prime }\right)\int_{R}d\eta \;e^{2i\left(p-p^{\prime }\right)\sqrt{qq^{\prime }}\eta }\tilde{F}\left[\eta \right]\bar{\tilde{G}\left[\eta \right]}\;.$$

### 4.5. Invertible W-H-like Affine Covariant Quantization

Trivially, if we impose in (71) the relation $\tilde{G}\left(\eta \right)={\bar{\tilde{F}\left(\eta \right)}}^{-1}$, then

(72) $$\mathrm{tr}\;\left(P_{q,p}^{\left(F\right)}P_{q^{\prime },p^{\prime }}^{\left(G\right)}\right)=2\pi \;\delta \left(q-q^{\prime }\right)\;\delta \left(p-p^{\prime }\right)\;.$$

This means that the quantization map is invertible. The simplest case is obtained for $\tilde{F}\left(\eta \right)=\tilde{G}\left(\eta \right)=1$ which corresponds to

(73) $$F\left(u\right)=\frac{1}{\sqrt{2}}\sqrt{u+\frac{1}{u}}\;.$$

We notice that the constraint F(1)=1 is verified. This solution gives an affine counterpart of the Wigner-Weyl transform since we need an unique function to build the quantization map and its inverse. However, we notice that the function *F* of (73) does not fulfill the boundedness condition $|u^{2}F\left(u\right)|\leq C$ which was requested at the beginning of this section. Therefore the operators $P_{q,p}^{\left(F\right)}$ involved in this case might be unbounded. In fact, this solution is a special case of a larger family of functions: $F_{\nu }\left(u\right)$ with

(74) $$F_{\nu }\left(u\right)={\left(\frac{1}{2}\left(u+u^{-1}\right)\right)}^{\nu +1/2}\;.$$

The “conjugate function” allowing to build the inverse map due to $F_{\nu }\left(u\right)$ is just $F_{-\nu }\left(u\right)$.

The boundedness condition $|u^{2}F_{\nu }\left(u\right)|\leq C$ is fulfilled only for ν≤−5/2. Therefore $F_{\nu }$ and $F_{-\nu }$ cannot fulfill this condition at once. However, if we assume ν≤−5/2 for the quantization mapping, then $F^{\prime \prime }\left(1\right)=\frac{3}{2}\left(\nu +1/2\right)<-3$. Therefore in that case the operator $A_{p^{2}}^{\left(F_{\nu }\right)}$ has a unique self-adjoint extension. We notice also that for ν=0 (our analogue of Wigner-Weyl) we obtain an attractive potential in $A_{p^{2}}^{\left(F\right)}$.

### 4.6. Discussion

Some Wigner-like and Weyl-like aspects of affine covariant quantization are presented in [11]. The calculations developed in Section 7 of [11] correspond to the simplest case F(u)=1 which corresponds to ν=−1/2 in our family $F_{\nu }$. This choice allows to reproduce in the affine framework the Wigner-Weyl properties $A_{f(q)}=f\left(Q\right)$ and $A_{f(p)}=f\left(P\right)$. However, in that case the inverse of the quantization mapping cannot be built using the same function (as noticed in Proposition 7.5 of [11]) and there exists different possible self-adjoint extensions of the quantized kinetic operator $A_{p^{2}}=P^{2}$ (as noticed below Equation (7.7) of [11]). Therefore this choice is not a complete analogue of the Wigner-Weyl map. In fact, a complete analogue of the Wigner-Weyl map does not exist in the affine framework. In general for ν≠−1/2 we fail to impose $A_{f(p)}=f\left(P\right)$, but for ν=0 we preserve the use of a unique function (operator) for the inverse map, while for ν<−5/2 we are able to uniquely specify the self-adjoint kinetic operator $A_{p^{2}}$.

## 5. Conclusions

Through the above specifications of covariant integral quantization, in their Wigner-Weyl-like restrictions, to two basic cases, the euclidean plane with its translational symmetry on one hand, the open half-plane with its affine symmetry on the other hand, we have provided an illustration of the crucial role of the Fourier transform, which is needed at each step of the calculations. With these generalizations of the Wigner-Weyl transform we have shown that the Weyl integral quantization, often thought of as the “best” option, has many interesting features shared by a wide panel of other integral quantizations.

We also think that similar features hold far beyond the two elementary symmetries which have been examined here. There exist many versions of the Wigner function or equivalent quasi-distribution for other groups, see for instance [39,40] for SU(n) and references therein. In the case of non-compact groups, particularly those which are semi-direct products of groups, the existence of square-integrability of the UIR requested by the resolution of the identity lying at the heart of the construction is in general not guaranteed. However, we think that it is possible to get round this issue if square-integrability of the UIR holds with respect to a subgroup. Related concepts and material on the restricted level of coherent states are found for instance in [41] and the chapters 7 and 8 of [10] with references therein.

As a final comment, the methods of quantization which have been exposed here are just a tiny part of a huge variety of ways of building quantum models from a unique classical one. We should always keep in our mind that mathematical models for physical systems are mainly effective, and the freedom one has in picking one specific model should be considered as an attractive feature rather than a drawback [42].

## Appendix A. Quantization of The Plane: Boundedness Of $P_{0}^{\left(F\right)}$

We prove the bounded character of the operator $P_{0}^{\left(F\right)}$ when *F* belongs to $L^{1}\left(R,du\right)⋂L^{1}\left(R,|u^{2}{-1/4|}^{-1/2}du\right)$. From the Riesz lemma it is sufficient to prove that $B\left(\phi ,\psi \right)=\langle \phi |P_{0}^{\left(F\right)}|\psi \rangle$ is a bounded bilinear form. Using (33) we have

(A1) $$\left|B\left(\phi ,\psi \right)\right|\leq \int_{R}|F\left(u\right)|du\int_{R}dz\;|\bar{\phi ((u+1/2)z)}|\;|\psi \left(\left(u-1/2\right)z\right)|\;,$$

Using Cauchy-Schwarz inequality and a change of variable we obtain

(A2) $$\int_{R}dz\;|\bar{\phi ((u+1/2)z)}|\;|\psi \left(\left(u-1/2\right)z\right)|\;\leq \frac{1}{\sqrt{|u^{2}-1/4|}}∥\phi ∥\;∥\psi ∥\;.$$

Therefore if *F* belongs to $L^{1}\left(R,du\right)⋂L^{1}\left(R,|u^{2}{-1/4|}^{-1/2}du\right)$ we have |B(ϕ,ψ)|≤C||ϕ||||ψ|| with $C=\int_{R}\left|F\left(u\right)|\;\right|u^{2}{-1/4|}^{-1/2}\;du$ and B(ϕ,ψ) is a bounded bilinear functional.

We notice that the same reasoning holds if we replace F(u)du by a positive measure dμ(u) such that $u\mapsto |u^{2}{-1/4|}^{-1/2}$ belongs to $L^{1}\left(R,d\mu \left(u\right)\right)$. This is in particular the case when we choose F(u)=δ(u) (Wigner-Weyl transform).

## Appendix B. Quantization of The Half-Plane: Boundedness of $P_{q,p}^{\left(F\right)}$

We prove the boundedness of the operator $P_{q,p}^{\left(F\right)}$ when $u\mapsto u^{2}F\left(u\right)$ is a bounded function. From the Riesz lemma it is sufficient to prove that $B\left(\phi ,\psi \right)=\langle \phi |P_{q,p}^{\left(F\right)}|\psi \rangle$ is a bounded bilinear form. From (58) B(ϕ,ψ) reads

(A3) $$B\left(\phi ,\psi \right)=\int_{R^{+}}dx\;\frac{2x}{q}F\left(x/q\right)\;\bar{\phi (x)}\;\psi \left(q^{2}/x\right)\;e^{ip(x-q^{2}/x)}\;.$$

Therefore we obtain:

(A4) $$\left|B\left(\phi ,\psi \right)\right|\leq 2\int_{R^{+}}dx\;\frac{x^{2}}{q^{2}}F\left(x/q\right)|\phi \left(x\right)|\;\frac{q}{x}\left|\psi \left(q^{2}/x\right)\right|\;.$$

Thus if $u\mapsto u^{2}F\left(u\right)$ is a bounded function with $|u^{2}F\left(u\right)|\leq C$ we have

(A5) $$\left|B\left(\phi ,\psi \right)\right|\leq 2C\int_{R^{+}}dx\;|\phi \left(x\right)|\;\frac{q}{x}\left|\psi \left(q^{2}/x\right)\right|\;.$$

Then using the Cauchy-Schwarz inequality and a change of variable in the integral involving $\left(q/x\right)\psi \left(q^{2}/x\right)$ we obtain:

(A6) |B(ϕ,ψ)|≤2C∥ϕ∥∥ψ∥.

We conclude that the operator $P_{q,p}^{\left(F\right)}$ is bounded.
